# Semaphorin 3A: A Potential Target for Low Back Pain

**DOI:** 10.3389/fnagi.2015.00216

**Published:** 2015-11-26

**Authors:** Pengbin Yin, Houchen Lv, Lihai Zhang, Licheng Zhang, Peifu Tang

**Affiliations:** Department of Orthopedics, Chinese PLA General HospitalBeijing, China

**Keywords:** low back pain, semaphorin 3A, neuropilins, pathological innervation, inter-vertebral disc degeneration

## Abstract

Low back pain is a common disorder. Pathological innervation and intervertebral disc degeneration are two major factors associated with this disease. Semaphorin 3A, originally known for its potent inhibiting effect on axonal outgrowth, is recently found to correlate with disease activity and histological features in some skeletal disorders. Based on its effects on innervation and vascularization, as well as enzyme secretion, we presume that semaphorin 3A may act as a potential target for low back pain.

## Introduction

Low back pain (LBP) is a common disorder. Around 60% of the adult population suffers from back discomfort at some point in their lifetime (Hoy et al., 2012; Campbell and Colvin, 2013). However, a definite pathological cause to this sickness is still unknown. Prior research has reported various potential factors, which may be associated with back pain, such as mechanical changes, low pH throughout the disc, disc degeneration, cytokines etc. Anatomically, it has been proposed that degenerative joint disease and intervertebral disc degeneration are the most common factors. In addition, pathological innervation into the degenerative intervertebral disc is said to be closely related with pain of intervertebral disc origin (Luoma et al., 2000; Cheung et al., 2009; García-Cosamalón et al., 2010; Miyagi et al., 2014). Thus, most therapeutic research on low back pain focuses on inhibiting neural invasion and delaying disc degradation (Mantyh, 2014; Vasiliadis et al., 2014; Sakai and Grad, 2015).

Semaphorin 3A, originally known for its potent inhibition of axonal outgrowth, has been found to play pivotal roles in several other systems (Luo et al., 1993; Barresi et al., 2009; Shim et al., 2013; van Gils et al., 2013). Notably, several recent studies have suggested that the expression of semaphorin 3A and its receptors (neuropilins) correlates with disease activity and histological features in some skeletal disorders (Hayashi et al., 2012; Negishi-Koga and Takayanagi, 2012; Fukuda et al., 2013; Takagawa et al., 2013). Previous research has demonstrated that semaphorin 3A is associated with innervation and vascularization, enzyme secretion, and cartilage development in skeletal tissues (Gomez et al., 2005; Okubo et al., 2011; Fukuda et al., 2013). These effects contribute to physiological and pathological alteration of the skeletal system. Further research also indicates the important role of enzyme secretion in disc degradation (Canbay et al., 2013; Xu et al., 2014). Since semaphorin 3A has been proven to be involved in both processes, we question that if there is any correlation between semaphorin 3A and low back pain.

## Hypothesis

Based on the effects of semaphorin 3A on innervation and vascularization, as well as on enzyme secretion in the skeletal system, coupled with the understanding of pathophysiology of low back pain, we hypothesize that semaphorin 3A may be a potential therapeutic target for low back pain.

## Evaluation of the hypothesis

It has been observed in both animal and human studies that with the progression of degeneration, sensory nerve fibers—which require a low level of chemical and mechanical stimuli to trigger pain—begin to innervate normally anural and avascular areas (Freemont et al., 1997; Miyagi et al., 2014). Research by Mantyh et al. showed that the administration of anti-nerve growth factors dramatically block the sprouting of these fibers, therefore significantly inhibited pain in mice (Mantyh et al., 2010). Therefore, an approach to inhibit pathological neural and vascular innervation in degenerative vertebral discs represents a new potential for pain management and treatment.

The mechanisms underlying the degeneration of intervertebral discs along with aging are complex, though MMP has been shown to play an important role. Research showed that the expression of MMPs is positively related to the severity of degeneration (Rutges et al., 2008; Xu et al., 2014). Based on their catabolic biological activities, the function of these enzymes is to maintain the integrity of the matrix by cooperating with other factors that perform anabolic activities (Le Maitre et al., 2007). However, in a pathological condition the balance disrupts and can lead to excessive degradation of disc components (Vo et al., 2013).

Semaphorin 3A, a prototypical class 3 secreted semaphorin, is a potent inhibitor of axonal outgrowth in a specific subset of sensory and sympathetic neurons and induces collapse of their growth cones. Research by Sotonye et al. showed that semaphorin 3A is highly expressed by healthy disc cells and decreased significantly in degenerate samples (Tolofari et al., 2010). Considering its inhibition of axonal outgrowth, semaphorin 3A may act as a biological barrier against neuronal ingrowth within healthy intervertebral disc. In addition, mRNA for semaphorin 3A receptors (neuropilins) was identified in healthy and degenerate tissues (Tolofari et al., 2010). Neuropilins have also been confirmed to bind to vascular endothelial growth factor (VEGF), which is a key regulator of normal and pathologic angiogenesis (Dai and Rabie, 2007). As VEGF and class 3 semaphorins compete for binding to neuropilins, reduction of semaphorin 3A may lead to increased binding of VEGF. Furthermore, binding of neuropilins to VEGF has been shown to result in promotion of the migration, proliferation, and tube formation of endothelial cells (Bates et al., 2003; Dai and Rabie, 2007; Roskoski, 2007; Staton et al., 2007). This process provides chemoattractive cues for vascular innervation, which may be associated with vascularization in degenerated intervertebral disc. All in all, semaphorin 3A is a potent inhibitor of both pathological innervation and vascular proliferation.

Research on lung cancer has further shown a negative correlation between protein expression levels of semaphorin 3A and MMP-14 (Zhou et al., 2014). In neurons, semaphorin 3A was also shown to consistently reduce MMP-3 expression and activity (Gonthier et al., 2009). Recent research on osteoarthritic cartilage has also shown that semaphorin 3A inhibited VEGF165-induced overexpression of MMPs (Okubo et al., 2011). These findings demonstrate the potential role of semaphorin 3A in negatively regulating MMP secretion. Although there is no direct evidence showing a relationship between the expression of MMPs and semaphorin 3A in patients with low back pain, the observations in other tissues provide some clues on the effects of semaphorin 3A on degenerative intervertebral discs.

Therefore, based on the potent effects of semaphorin 3A on repelling nerve ingrowth and vascular proliferation, as well as its negative regulation of MMPs, we hypothesize that semaphorin 3A may be a potential therapeutic target for low back pain.

## Author contributions

All of the authors meet all 4 of the requirements as stipulated in the Guide for Authors. Substantial contribution to the concept and design of this study: Pengbin Yin, Peifu Tang, and Licheng Zhang; literature retrieval: Licheng Zhang, Houchen Lv; and manuscript drafting: Pengbin Yin.

## Funding

This research project was supported by a grant from the National Natural Science Foundation of China (81401809).

### Conflict of interest statement

The authors declare that the research was conducted in the absence of any commercial or financial relationships that could be construed as a potential conflict of interest.

## References

[B1] Barresi V. Vitarelli E. Cerasoli S. (2009). Semaphorin3A immunohistochemical expression in human meningiomas: correlation with the microvessel density. Virch. Arch. 454, 563–571. 10.1007/s00428-009-0757-3 19296128

[B2] Bates D. Taylor G. I. Minichiello J. Farlie P. Cichowitz A. Watson N.. (2003). Neurovascular congruence results from a shared patterning mechanism that utilizes Semaphorin3A and Neuropilin-1. Dev. Biol. 255, 77–98. 10.1016/S0012-1606(02)00045-3 12618135

[B3] Campbell J. Colvin L. A. (2013). Management of low back pain. BMJ 347:f3148. 10.1136/bmj.f3148 24308922

[B4] Canbay S. Turhan N. Bozkurt M. Arda K. Caglar S. (2013). Correlation of matrix metalloproteinase-3 expression with patient age, magnetic resonance imaging and histopathological grade in lumbar disc degeneration. Turk. Neurosurg. 23, 427–433. 10.5137/1019-5149.JTN.7459-12.0 24101259

[B5] Cheung K. M. Karppinen J. Chan D. Ho D. W. Song Y. Q. Sham P.. (2009). Prevalence and pattern of lumbar magnetic resonance imaging changes in a population study of one thousand forty-three individuals. Spine (Phila Pa 1976) 34, 934–940. 10.1097/BRS.0b013e3181a01b3f 19532001

[B6] Dai J. Rabie A. B. (2007). VEGF: an essential mediator of both angiogenesis and endochondral ossification. J. Dent. Res. 86, 937–950. 10.1177/154405910708601006 17890669

[B7] Freemont A. J. Peacock T. E. Goupille P. Hoyland J. A. O'Brien J. Jayson M. I. (1997). Nerve ingrowth into diseased intervertebral disc in chronic back pain. Lancet 350, 178–181. 10.1016/S0140-6736(97)02135-1 9250186

[B8] Fukuda T. Takeda S. Xu R. Ochi H. Sunamura S. Sato T.. (2013). Sema3A regulates bone-mass accrual through sensory innervations. Nature 497, 490–493. 10.1038/nature12115 23644455

[B9] García-Cosamalón J. del Valle M. E. Calavia M. G. García-Suárez O. López-Muñiz A. Otero J.. (2010). Intervertebral disc, sensory nerves and neurotrophins: who is who in discogenic pain? J. Anat. 217, 1–15. 10.1111/j.1469-7580.2010.01227.x 20456524 PMC2913007

[B10] Gomez C. Burt-Pichat B. Mallein-Gerin F. Merle B. Delmas P. D. Skerry T. M.. (2005). Expression of Semaphorin-3A and its receptors in endochondral ossification: potential role in skeletal development and innervation. Dev. Dynam. 234, 393–403. 10.1002/dvdy.20512 16145665

[B11] Gonthier B. Koncina E. Satkauskas S. Perraut M. Roussel G. Aunis D.. (2009). A PKC-dependent recruitment of MMP-2 controls semaphorin-3A growth-promoting effect in cortical dendrites. PLoS ONE 4:e5099. 10.1371/journal.pone.0005099 19352510 PMC2663036

[B12] Hayashi M. Nakashima T. Takayanagi H. (2012). The regulatory mechanisms of bone metabolism by semaphorin. Clin. Calcium. 22, 1693–1699. 23103813

[B13] Hoy D. Bain C. Williams G. March L. Brooks P. Blyth F.. (2012). A systematic review of the global prevalence of low back pain. Arthritis Rheum. 64, 2028–2037. 10.1002/art.34347 22231424

[B14] Le Maitre C. L. Pockert A. Buttle D. J. Freemont A. J. Hoyland J. A. (2007). Matrix synthesis and degradation in human intervertebral disc degeneration. Biochem. Soc. Trans. 35(Pt 4), 652–655. 10.1042/BST0350652 17635113

[B15] Luo Y. Raible D. Raper J. A. (1993). Collapsin: a protein in brain that induces the collapse and paralysis of neuronal growth cones. Cell 75, 217–227. 10.1016/0092-8674(93)80064-L 8402908

[B16] Luoma K. Riihimäki H. Luukkonen R. Raininko R. Viikari-Juntura E. Lamminen A. (2000). Low back pain in relation to lumbar disc degeneration. Spine (Phila Pa 1976) 25, 487–492. 10.1097/00007632-200002150-00016 10707396

[B17] Mantyh P. W. (2014). The neurobiology of skeletal pain. Eur. J. Neurosci. 39, 508–519. 10.1111/ejn.12462 24494689 PMC4453827

[B18] Mantyh W. G. Jimenez-Andrade J. M. Stake J. I. Bloom A. P. Kaczmarska M. J. Taylor R. N.. (2010). Blockade of nerve sprouting and neuroma formation markedly attenuates the development of late stage cancer pain. Neuroscience 171, 588–598. 10.1016/j.neuroscience.2010.08.056 20851743 PMC2992976

[B19] Miyagi M. Millecamps M. Danco A. T. Ohtori S. Takahashi K. Stone L. S. (2014). Increased innervation and sensory nervous system plasticity in a mouse model of low back pain due to intervertebral disc degeneration. Spine (Phila Pa 1976). 39, 1345–1354. 10.1097/BRS.0000000000000334 24718079

[B20] Negishi-Koga T. Takayanagi H. (2012). Bone cell communication factors and Semaphorins. Bonekey Rep. 1:183. 10.1038/bonekey.2012.183 24171101 PMC3810552

[B21] Okubo M. Kimura T. Fujita Y. Mochizuki S. Niki Y. Enomoto H.. (2011). Semaphorin 3A is expressed in human osteoarthritic cartilage and antagonizes vascular endothelial growth factor 165-promoted chondrocyte migration: an implication for chondrocyte cloning. Arthritis Rheum. 63, 3000–3009. 10.1002/art.30482 21953086

[B22] Roskoski R. Jr. (2007). Vascular endothelial growth factor (VEGF) signaling in tumor progression. Crit. Rev. Oncol. Hematol. 62, 179–213. 10.1016/j.critrevonc.2007.01.006 17324579

[B23] Rutges J. P. Kummer J. A. Oner F. C. Verbout A. J. Castelein R. J. Roestenburg H. J.. (2008). Increased MMP-2 activity during intervertebral disc degeneration is correlated to MMP-14 levels. J. Pathol. 214, 523–530. 10.1002/path.2317 18200629

[B24] Sakai D. Grad S. (2015). Advancing the cellular and molecular therapy for intervertebral disc disease. Adv. Drug Deliv. Rev. 84, 159–171. 10.1016/j.addr.2014.06.009 24993611

[B25] Shim E. J. Chun E. Kang H. R. Cho S. H. Min K. U. Park H. W. (2013). Expression of semaphorin 3A and neuropilin 1 in asthma. J. Korean Med. Sci. 28, 1435–1442. 10.3346/jkms.2013.28.10.1435 24133346 PMC3792596

[B26] Staton C. A. Kumar I. Reed M. W. Brown N. J. (2007). Neuropilins in physiological and pathological angiogenesis. J. Pathol. 212, 237–248. 10.1002/path.2182 17503412

[B27] Takagawa S. Nakamura F. Kumagai K. Nagashima Y. Goshima Y. Saito T. (2013). Decreased semaphorin3A expression correlates with disease activity and histological features of rheumatoid arthritis. BMC Musculoskelet. Disord. 14:40. 10.1186/1471-2474-14-40 23343469 PMC3558329

[B28] Tolofari S. K. Richardson S. M. Freemont A. J. Hoyland J. A. (2010). Expression of semaphorin 3A and its receptors in the human intervertebral disc: potential role in regulating neural ingrowth in the degenerate intervertebral disc. Arthritis Res. Ther. 12:R1. 10.1186/ar2898 20051117 PMC2875625

[B29] van Gils J. M. Ramkhelawon B. Fernandes L. Stewart M. C. Guo L. Seibert T.. (2013). Endothelial expression of guidance cues in vessel wall homeostasis dysregulation under proatherosclerotic conditions. Arterioscler. Thromb. Vasc. Biol. 33, 911–919. 10.1161/ATVBAHA.112.301155 23430612 PMC3647028

[B30] Vasiliadis E. S. Pneumaticos S. G. Evangelopoulos D. S. Papavassiliou A. G. (2014). Biologic treatment of mild and moderate intervertebral disc degeneration. Mol. Med. 20, 400–409. 10.2119/molmed.2014.00145 25171110 PMC4212014

[B31] Vo N. V. Hartman R. A. Yurube T. Jacobs L. J. Sowa G. A. Kang J. D. (2013). Expression and regulation of metalloproteinases and their inhibitors in intervertebral disc aging and degeneration. Spine J. 13, 331–341. 10.1016/j.spinee.2012.02.027 23369495 PMC3637842

[B32] Xu H. Mei Q. Xu B. Liu G. Zhao J. (2014). Expression of matrix metalloproteinases is positively related to the severity of disc degeneration and growing age in the East Asian lumbar disc herniation patients. Cell Biochem. Biophys. 70, 1219–1225. 10.1007/s12013-014-0045-y 24874308

[B33] Zhou H. Wu A. Fu W. Lv Z. Zhang Z. (2014). Significance of semaphorin-3A and MMP-14 protein expression in non-small cell lung cancer. Oncol. Lett. 7, 1395–1400. 10.3892/ol.2014.1920 24765144 PMC3997704

